# Bis(1,10-phenanthrolin-1-ium) 9,10-di­oxo-9,10-dihydroanthracene-1,5-disul­fonate hexa­hydrate

**DOI:** 10.1107/S1600536810053456

**Published:** 2011-01-08

**Authors:** Jia Jia

**Affiliations:** a Department of Chemistry, Baicheng Normal College, Baicheng, Jilin 137000, People’s Republic of China

## Abstract

The title hydrated molecular salt, 2C_12_H_9_N_2_^+^·C_14_H_6_O_8_S_2_^2−^·6H_2_O, consists of 1,10-phenanthrolinium (phen-H^+^) cations, anthraquinone-1,5-disulfonate (AQDS^2−^) anions, which occupy a centre of inversion, and water molecules of crystal­lization. In the crystal, a supra­molecular network structure is formed *via* N—H⋯O and O—H⋯O hydrogen bonds and weak C—H⋯O and π–π stacking inter­actions [centroid–centroid distances = 3.651 (6) and 3.545 (8) Å].

## Related literature

For examples of multiple binding of 1,5-naphthalene­disulf­onate, see: Gao *et al.* (2005 ▶); Voogt & Blanch (2005 ▶). For the crystal structure of *o*-phenanthroline, see: Nishigaki *et al.* (1978 ▶). For the changes in protonated *o*-phenanthroline, see: Shriver *et al.* (1994 ▶). For weakly N—H⋯O hydrogen-bonded sulfonate ligands, see: Onoda *et al.* (2001 ▶). For graph-set analysis of hydrogen-bond patterns, see: Bernstein *et al.* (1995 ▶). 
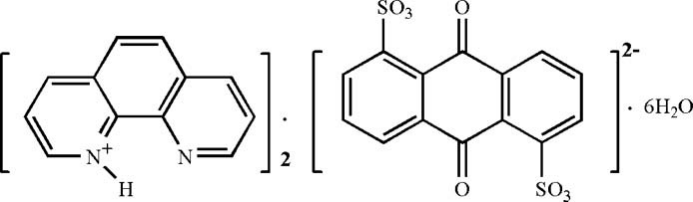


## Experimental

### 

#### Crystal data

2C_12_H_9_N_2_^+^·C_14_H_6_O_8_S_2_^2−^·6H_2_O
*M* *_r_* = 836.83
Triclinic, 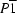

*a* = 10.0439 (6) Å
*b* = 10.1978 (6) Å
*c* = 11.1070 (6) Å
α = 111.591 (1)°
β = 98.848 (1)°
γ = 111.234 (1)°
*V* = 930.34 (9) Å^3^
*Z* = 1
Mo *K*α radiation
μ = 0.22 mm^−1^
*T* = 296 K
0.24 × 0.22 × 0.20 mm

#### Data collection

Bruker APEXII CCD area-detector diffractometer
Absorption correction: multi-scan (*SADABS*; Sheldrick, 1996 ▶) *T*_min_ = 0.782, *T*_max_ = 1.000
5035 measured reflections
3499 independent reflections
2964 reflections with *I* > 2σ(*I*)
*R*_int_ = 0.009

#### Refinement

*R*[*F* ^2^ > 2σ(*F* ^2^)] = 0.047
*wR*(*F* ^2^) = 0.100
*S* = 1.06
3499 reflections
265 parameters
H atoms treated by a mixture of independent and constrained refinement
Δρ_max_ = 0.42 e Å^−3^
Δρ_min_ = −0.39 e Å^−3^

### 

Data collection: *APEX2* (Bruker, 2003 ▶); cell refinement: *SAINT* (Bruker, 2001 ▶); data reduction: *SAINT*; program(s) used to solve structure: *SHELXS97* (Sheldrick, 2008 ▶); program(s) used to refine structure: *SHELXL97* (Sheldrick, 2008 ▶); molecular graphics: *SHELXTL* (Sheldrick, 2008 ▶) and *DIAMOND* (Brandenburg & Berndt, 1999 ▶); software used to prepare material for publication: *SHELXL97* and *PLATON* (Spek, 2009 ▶).

## Supplementary Material

Crystal structure: contains datablocks I, global. DOI: 10.1107/S1600536810053456/si2318sup1.cif

Structure factors: contains datablocks I. DOI: 10.1107/S1600536810053456/si2318Isup2.hkl

Additional supplementary materials:  crystallographic information; 3D view; checkCIF report

## Figures and Tables

**Table 1:** Hydrogen-bond geometry (Å, °)

*D*—H⋯*A*	*D*—H	H⋯*A*	*D*⋯*A*	*D*—H⋯*A*
N1—H1*A*⋯O5	0.87 (3)	1.91 (3)	2.713 (3)	153 (3)
O5—H5*A*⋯O2^i^	0.82	1.97	2.776 (3)	168
O5—H5*B*⋯O2	0.82	2.19	2.931 (3)	150
O5—H5*B*⋯O4	0.82	2.48	3.131 (3)	138
O6—H6*A*⋯O3^ii^	0.82	1.96	2.775 (3)	170
O6—H6*B*⋯O1^iii^	0.82	1.92	2.739 (3)	172
O7—H7*A*⋯O6	0.82	1.97	2.774 (4)	167
O7—H7*B*⋯O7^iv^	0.82	2.20	3.021 (6)	178
O7—H7*C*⋯O5	0.82	2.48	3.301 (4)	179
C2—H2*A*⋯O7	0.93	2.39	3.300 (4)	167
C4—H4*A*⋯O1^v^	0.93	2.58	3.418 (4)	151
C6—H6⋯O4^v^	0.93	2.54	3.350 (3)	145
C18—H18⋯O3^vi^	0.93	2.51	3.357 (4)	152

Symmetry codes: (i) 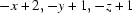; (ii) 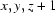; (iii) 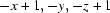; (iv) 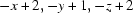; (v) 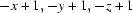; (vi) 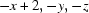.

**Fig. 1.**
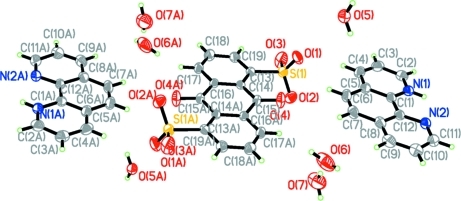
The molecular structure of (I) drawn with 30% probability displacement ellipsoids.[symmetry code: 2 – x, 1 – y, 1 – z.]

**Fig. 2.**
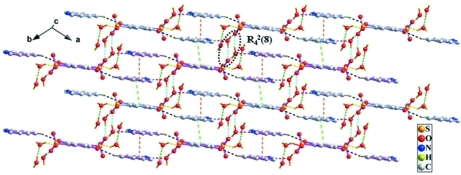
A perspective view of the two-dimensional structure. Hydrogen bonds and π-π stacking interactions are indicated by dashed lines. H atoms not involved in the hydrogen bonds shown have been omitted.

## supplementary crystallographic information

### Comment

The anthraquinone-1,5-disulfonate (AQDS ^2-^) possesses six sulfonate O atoms and has been also employed either as a counter ion, forming extensive hydrogen-bonding interaction with water molecules or as a ligand with multiple binding sites available to construct coordination polymers with varying dimensionalities (Gao *et al.*, 2005; Voogt & Blanch, 2005). Hydrogen bonding patterns involving sulfonate in biological systems and metal complexes are of current interest (Onoda *et al.*, 2001). In the molecular structure of the title cocrystal, (phen-H^+^)_2_(AQDS^2-^).6H_2_O (I), the dianion exhibits crystallographic inversion symmetry (Fig. 1). A supramolecular network structure is formed *via* N—H···O and O—H···O hydrogen bonds, and weak C—H···O and π-π stacking interactions (Fig. 2 and Table 1) support the stability of the crystal packing.

The phen-H^+^ cation is planar, with deviations of the non-H atoms from the least-squares plane of less than 0.021 (1) Å in the phen-H^+^ cation consisting of N1, N2, C1 to C12. The angle between the planar cation and planar anion in the asymmetric unit is 2.133 (3)°. Compared with the neutral base, 1,10-phenanthroline (Nishigaki *et al.*, 1978), the bond distances in the phen-H^+^ cations show no significant differences, but some internal bond angles vary significantly, at the protonated N1 atom angle C2—N1—C1 = 123.1 (3)° and angle at neutral N2, C11—N2—C12 = 116.6 (3)°, which makes a difference of 6.5 (3)°. This is due to the formation of N—H bonds, which have a less repulsive effect on the N—C bonds than the lone electron pair (Shriver *et al.*, 1994).

The phen-H^+^ cations and AQDS ^2-^ anions are held together by weak hydrogen bonds C6—H6···O4^v^. Such adjacent units are assembled into 1-D chain through π-π stacking interactions between the adjacent phen-H^+^ rings (Fig. 2). *Cg*3···*Cg*4^v^ distance is 3.651 (6) Å, the perpendicular distance between the inversion-related planes (therefore the dihedral angle is zero) is 3.395 (6) Å. *Cg*3 is the centroid of the ring (N1, C2, C3, C4, C5, C1), *Cg*4 is the centroid of the ring (N2, C12, C8, C9, C10, C11), and the symmetry code v = 1 - *x*, 1 - *y*, 1 - *z*. Additionally, the adjacent 1-D chains are extended into a two-dimensional structure also by *π*-*π* stacking interaction between the anthraquinone ring and phen-H^+^ ring. *Cg*2···*Cg*4^iii^ distance is 3.545 (8) Å, *Cg*2 is the centroid of the ring (C15, C14, C16, C15A, C14A, C16A), and the symmetry code iii = 1 - *x*, - *y*, 1 - *z*.

Meanwhile, the lattice water molecules are anchored on the layer *via* hydrogen bonds (O5—H5A···O2^i^, O5—H5B···O2), resulting in the hydrogen-bonded ring motifs of *R*_4_^2^ (8) (Bernstein *et al.*, 1995). Such rings are linked to phen-H^+^ cations by hydrogen bond N1—H1A···O5. Dimeric water molecules are linked *via* O7—H7A···O6, and are connected to the sulfonate oxygen O3 atom by O6—H6A···O3^ii^ (Fig. 2). The hydrogen-bond geometries and symmetry codes are listed in Table 1. In conclusion, this work indicates that anthraquinone-1,5-disulfonate is also a good participant in hydrogen-bonding and π-π stacking networks for the formation of acid-base molecular cocrystals.

### Experimental

A mixture of disodium anthraquinone-1,5-disulfonate (0.2 mmol, 82.4 mg), FeCl_3_.6H_2_O (0.2 mmol, 54.06 mg), 1,10-phenanthroline (0.2 mmol, 39.64 mg), and H_2_O (10 mL) was sealed in a 23 ml teflonlined stainless steel vessel under autogenous pressure; the vessel was heated to 150°C for 4 days and then cooled to room temperature at a rate of 2.6°C/h. The reaction mixture was filtered and yellow block-shaped crystals were collected by slow evaporation of the solvent.

### Refinement

H atoms were located in difference maps, but were subsequently placed in calculated positions and treated as riding, with C···H = 0.930Å and N···H = 0.860 Å. All H atoms were allocated displacement parameters related to those of their parent atoms [*U*_iso_(H) = 1.2Ueq(C, N)].

### Crystal data

**Table:**

2C_12_H_9_N_2_^+^·C_14_H_6_O_8_S_2_^2^^−^·6H_2_O	*Z* = 1
*M**_r_* = 836.83	*F*(000) = 436
Triclinic, *P*1	*D*_x_ = 1.494 Mg m^−^^3^
Hall symbol: -P 1	Mo *K*α radiation, λ = 0.71073 Å
*a* = 10.0439 (6) Å	Cell parameters from 3063 reflections
*b* = 10.1978 (6) Å	θ = 2.4–27.6°
*c* = 11.1070 (6) Å	µ = 0.22 mm^−^^1^
α = 111.591 (1)°	*T* = 296 K
β = 98.848 (1)°	Block, yellow
γ = 111.234 (1)°	0.24 × 0.22 × 0.20 mm
*V* = 930.34 (9) Å^3^	

### Data collection

**Table:**

Bruker APEXII CCD area-detector diffractometer	3499 independent reflections
Radiation source: fine-focus sealed tube	2964 reflections with *I* > 2σ(*I*)
graphite	*R*_int_ = 0.009
φ and ω scans	θ_max_ = 25.7°, θ_min_ = 2.1°
Absorption correction: multi-scan (*SADABS*; Sheldrick, 1996)	*h* = −12→9
*T*_min_ = 0.782, *T*_max_ = 1.000	*k* = −11→12
5035 measured reflections	*l* = −11→13

### Refinement

**Table:**

Refinement on *F*^2^	Primary atom site location: structure-invariant direct methods
Least-squares matrix: full	Secondary atom site location: difference Fourier map
*R*[*F*^2^ > 2σ(*F*^2^)] = 0.047	Hydrogen site location: inferred from neighbouring sites
*wR*(*F*^2^) = 0.100	H atoms treated by a mixture of independent and constrained refinement
*S* = 1.06	*w* = 1/[σ^2^(*F*_o_^2^) + (0.*P*)^2^ + 1.1*P*] where *P* = (*F*_o_^2^ + 2*F*_c_^2^)/3
3499 reflections	(Δ/σ)_max_ < 0.001
265 parameters	Δρ_max_ = 0.42 e Å^−^^3^
0 restraints	Δρ_min_ = −0.39 e Å^−^^3^

### Special details

**Table:**

Geometry. All e.s.d.'s (except the e.s.d. in the dihedral angle between two l.s. planes) are estimated using the full covariance matrix. The cell e.s.d.'s are taken into account individually in the estimation of e.s.d.'s in distances, angles and torsion angles; correlations between e.s.d.'s in cell parameters are only used when they are defined by crystal symmetry. An approximate (isotropic) treatment of cell e.s.d.'s is used for estimating e.s.d.'s involving l.s. planes.
Refinement. Refinement of *F*^2^ against ALL reflections. The weighted *R*-factor *wR* and goodness of fit *S* are based on *F*^2^, conventional *R*-factors *R* are based on *F*, with *F* set to zero for negative *F*^2^. The threshold expression of *F*^2^ > σ(*F*^2^) is used only for calculating *R*-factors(gt) *etc*. and is not relevant to the choice of reflections for refinement. *R*-factors based on *F*^2^ are statistically about twice as large as those based on *F*, and *R*- factors based on ALL data will be even larger.

### Fractional atomic coordinates and isotropic or equivalent isotropic displacement parameters (Å^2^)

**Table:**

	*x*	*y*	*z*	*U*_iso_*/*U*_eq_	Occ. (<1)
N1	0.7261 (2)	0.6318 (3)	0.6490 (2)	0.0508 (5)	
H1A	0.777 (3)	0.583 (3)	0.616 (3)	0.061*	
N2	0.7525 (2)	0.5796 (3)	0.3953 (2)	0.0529 (5)	
C1	0.6554 (3)	0.6801 (3)	0.5725 (3)	0.0473 (6)	
C2	0.7199 (3)	0.6540 (3)	0.7734 (3)	0.0614 (7)	
H2A	0.7702	0.6189	0.8220	0.074*	
C3	0.6388 (4)	0.7293 (4)	0.8305 (3)	0.0678 (8)	
H3A	0.6351	0.7461	0.9179	0.081*	
C4	0.5642 (3)	0.7788 (3)	0.7575 (3)	0.0654 (8)	
H4A	0.5086	0.8285	0.7953	0.078*	
C5	0.5701 (3)	0.7558 (3)	0.6260 (3)	0.0547 (7)	
C6	0.4937 (3)	0.8035 (3)	0.5441 (4)	0.0657 (8)	
H6	0.4366	0.8533	0.5779	0.079*	
C7	0.5030 (3)	0.7776 (3)	0.4189 (4)	0.0661 (8)	
H7	0.4522	0.8101	0.3678	0.079*	
C8	0.5896 (3)	0.7008 (3)	0.3618 (3)	0.0536 (7)	
C9	0.6022 (3)	0.6695 (3)	0.2310 (3)	0.0643 (8)	
H9A	0.5532	0.6992	0.1756	0.077*	
C10	0.6866 (4)	0.5954 (4)	0.1856 (3)	0.0652 (8)	
H10A	0.6953	0.5735	0.0990	0.078*	
C11	0.7599 (3)	0.5531 (4)	0.2714 (3)	0.0610 (7)	
H11A	0.8174	0.5030	0.2392	0.073*	
C12	0.6670 (3)	0.6521 (3)	0.4392 (3)	0.0469 (6)	
S1	0.78814 (8)	0.11641 (9)	0.22241 (7)	0.05292 (19)	
O1	0.6380 (2)	0.0104 (3)	0.2065 (3)	0.0829 (7)	
O2	0.8478 (2)	0.2720 (2)	0.3366 (2)	0.0633 (5)	
O3	0.8023 (3)	0.1250 (4)	0.0977 (2)	0.1002 (9)	
O4	0.7954 (2)	0.1068 (3)	0.4845 (2)	0.0661 (6)	
C13	0.9069 (3)	0.0280 (3)	0.2540 (2)	0.0395 (5)	
C14	0.9501 (2)	0.0149 (3)	0.3743 (2)	0.0346 (5)	
C15	0.8932 (3)	0.0643 (3)	0.4908 (2)	0.0383 (5)	
C16	1.0474 (2)	−0.0535 (3)	0.3832 (2)	0.0365 (5)	
C17	1.0957 (3)	−0.1127 (3)	0.2735 (3)	0.0506 (6)	
H17	1.1599	−0.1583	0.2805	0.061*	
C18	1.0489 (3)	−0.1042 (4)	0.1554 (3)	0.0602 (8)	
H18	1.0788	−0.1465	0.0812	0.072*	
C19	0.9570 (3)	−0.0324 (3)	0.1471 (3)	0.0524 (6)	
H19	0.9281	−0.0245	0.0674	0.063*	
O5	0.8925 (2)	0.4707 (2)	0.6231 (2)	0.0661 (6)	
H5A	0.9758	0.5440	0.6439	0.099*	
H5B	0.8657	0.3892	0.5524	0.099*	
O6	0.6538 (3)	0.2203 (3)	0.9409 (3)	0.1278 (13)	
H6A	0.6893	0.1913	0.9919	0.192*	
H6B	0.5678	0.1470	0.9015	0.192*	
O7	0.8515 (4)	0.4711 (3)	0.9125 (3)	0.1289 (12)	
H7A	0.7860	0.3911	0.9080	0.193*	
H7B	0.9314	0.4878	0.9619	0.193*	0.50
H7C	0.8613	0.4715	0.8406	0.193*	0.50

### Atomic displacement parameters (Å^2^)

**Table:**

	*U*^11^	*U*^22^	*U*^33^	*U*^12^	*U*^13^	*U*^23^
N1	0.0456 (13)	0.0436 (12)	0.0565 (14)	0.0198 (10)	0.0116 (10)	0.0190 (11)
N2	0.0491 (13)	0.0509 (13)	0.0583 (14)	0.0265 (11)	0.0134 (11)	0.0228 (11)
C1	0.0359 (13)	0.0337 (12)	0.0602 (16)	0.0118 (10)	0.0078 (11)	0.0169 (12)
C2	0.0577 (18)	0.0542 (17)	0.0579 (18)	0.0174 (14)	0.0096 (14)	0.0233 (14)
C3	0.0648 (19)	0.0566 (18)	0.0622 (19)	0.0178 (16)	0.0222 (16)	0.0171 (15)
C4	0.0542 (17)	0.0481 (16)	0.077 (2)	0.0190 (14)	0.0266 (16)	0.0151 (15)
C5	0.0419 (14)	0.0389 (14)	0.0711 (19)	0.0155 (12)	0.0154 (13)	0.0173 (13)
C6	0.0515 (17)	0.0521 (17)	0.094 (2)	0.0305 (14)	0.0218 (16)	0.0285 (17)
C7	0.0518 (17)	0.0539 (17)	0.095 (2)	0.0287 (14)	0.0111 (16)	0.0376 (17)
C8	0.0426 (14)	0.0402 (14)	0.0718 (19)	0.0154 (12)	0.0078 (13)	0.0275 (13)
C9	0.0605 (18)	0.0559 (17)	0.076 (2)	0.0212 (15)	0.0093 (15)	0.0398 (16)
C10	0.0660 (19)	0.0627 (19)	0.0659 (19)	0.0239 (16)	0.0191 (15)	0.0350 (16)
C11	0.0562 (17)	0.0614 (18)	0.0654 (19)	0.0291 (15)	0.0193 (14)	0.0271 (15)
C12	0.0368 (13)	0.0355 (13)	0.0595 (16)	0.0132 (11)	0.0079 (11)	0.0196 (12)
S1	0.0617 (4)	0.0595 (4)	0.0475 (4)	0.0369 (4)	0.0117 (3)	0.0279 (3)
O1	0.0439 (12)	0.0733 (15)	0.1110 (19)	0.0275 (11)	0.0012 (12)	0.0309 (14)
O2	0.0819 (14)	0.0497 (11)	0.0684 (13)	0.0355 (11)	0.0266 (11)	0.0308 (10)
O3	0.161 (3)	0.153 (3)	0.0635 (14)	0.122 (2)	0.0502 (16)	0.0712 (16)
O4	0.0783 (14)	0.1073 (17)	0.0764 (14)	0.0750 (14)	0.0483 (12)	0.0644 (13)
C13	0.0372 (12)	0.0404 (12)	0.0392 (12)	0.0173 (10)	0.0100 (10)	0.0182 (10)
C14	0.0320 (11)	0.0330 (11)	0.0369 (12)	0.0143 (9)	0.0106 (9)	0.0151 (9)
C15	0.0385 (12)	0.0396 (12)	0.0464 (13)	0.0227 (10)	0.0181 (10)	0.0229 (11)
C16	0.0356 (12)	0.0370 (12)	0.0375 (12)	0.0179 (10)	0.0138 (9)	0.0160 (10)
C17	0.0522 (15)	0.0625 (17)	0.0479 (15)	0.0370 (14)	0.0225 (12)	0.0228 (13)
C18	0.0671 (19)	0.083 (2)	0.0429 (15)	0.0455 (17)	0.0283 (14)	0.0256 (15)
C19	0.0560 (16)	0.0664 (18)	0.0388 (13)	0.0302 (14)	0.0160 (12)	0.0258 (13)
O5	0.0582 (12)	0.0608 (12)	0.0744 (14)	0.0309 (10)	0.0177 (10)	0.0243 (11)
O6	0.0916 (19)	0.0847 (18)	0.170 (3)	0.0179 (15)	−0.0330 (19)	0.074 (2)
O7	0.123 (2)	0.091 (2)	0.150 (3)	0.0416 (19)	−0.005 (2)	0.061 (2)

### Geometric parameters (Å, °)

**Table:**

N1—C2	1.330 (4)	S1—O1	1.439 (2)
N1—C1	1.359 (3)	S1—O2	1.443 (2)
N1—H1A	0.87 (3)	S1—O3	1.445 (2)
N2—C11	1.320 (4)	S1—C13	1.801 (2)
N2—C12	1.359 (3)	O4—C15	1.211 (3)
C1—C5	1.408 (4)	C13—C19	1.384 (3)
C1—C12	1.433 (4)	C13—C14	1.409 (3)
C2—C3	1.382 (4)	C14—C16	1.402 (3)
C2—H2A	0.9300	C14—C15	1.487 (3)
C3—C4	1.364 (4)	C15—C16^i^	1.494 (3)
C3—H3A	0.9300	C16—C17	1.391 (3)
C4—C5	1.406 (4)	C16—C15^i^	1.494 (3)
C4—H4A	0.9300	C17—C18	1.369 (4)
C5—C6	1.427 (4)	C17—H17	0.9300
C6—C7	1.341 (4)	C18—C19	1.381 (4)
C6—H6	0.9300	C18—H18	0.9300
C7—C8	1.439 (4)	C19—H19	0.9300
C7—H7	0.9300	O5—H5A	0.8200
C8—C9	1.405 (4)	O5—H5B	0.8200
C8—C12	1.406 (3)	O6—H6A	0.8201
C9—C10	1.363 (4)	O6—H6B	0.8200
C9—H9A	0.9300	O7—H7A	0.8199
C10—C11	1.398 (4)	O7—H7B	0.8200
C10—H10A	0.9300	O7—H7C	0.8200
C11—H11A	0.9300		
			
C2—N1—C1	123.0 (3)	C10—C11—H11A	117.9
C2—N1—H1A	117.5 (19)	N2—C12—C8	123.9 (3)
C1—N1—H1A	119.5 (19)	N2—C12—C1	117.7 (2)
C11—N2—C12	116.6 (2)	C8—C12—C1	118.4 (2)
N1—C1—C5	118.8 (3)	O1—S1—O2	113.37 (14)
N1—C1—C12	120.0 (2)	O1—S1—O3	113.36 (17)
C5—C1—C12	121.2 (2)	O2—S1—O3	111.51 (15)
N1—C2—C3	120.1 (3)	O1—S1—C13	106.06 (12)
N1—C2—H2A	119.9	O2—S1—C13	108.18 (11)
C3—C2—H2A	119.9	O3—S1—C13	103.59 (12)
C4—C3—C2	119.3 (3)	C19—C13—C14	119.0 (2)
C4—C3—H3A	120.3	C19—C13—S1	114.84 (18)
C2—C3—H3A	120.3	C14—C13—S1	126.17 (18)
C3—C4—C5	121.0 (3)	C16—C14—C13	118.6 (2)
C3—C4—H4A	119.5	C16—C14—C15	117.95 (19)
C5—C4—H4A	119.5	C13—C14—C15	123.4 (2)
C4—C5—C1	117.7 (3)	O4—C15—C14	122.0 (2)
C4—C5—C6	123.7 (3)	O4—C15—C16^i^	118.9 (2)
C1—C5—C6	118.6 (3)	C14—C15—C16^i^	119.07 (19)
C7—C6—C5	121.0 (3)	C17—C16—C14	120.7 (2)
C7—C6—H6	119.5	C17—C16—C15^i^	116.5 (2)
C5—C6—H6	119.5	C14—C16—C15^i^	122.7 (2)
C6—C7—C8	121.6 (3)	C18—C17—C16	120.1 (2)
C6—C7—H7	119.2	C18—C17—H17	119.9
C8—C7—H7	119.2	C16—C17—H17	119.9
C9—C8—C12	116.7 (3)	C17—C18—C19	119.7 (2)
C9—C8—C7	124.0 (3)	C17—C18—H18	120.2
C12—C8—C7	119.3 (3)	C19—C18—H18	120.2
C10—C9—C8	119.8 (3)	C18—C19—C13	121.8 (2)
C10—C9—H9A	120.1	C18—C19—H19	119.1
C8—C9—H9A	120.1	C13—C19—H19	119.1
C9—C10—C11	118.9 (3)	H5A—O5—H5B	117.0
C9—C10—H10A	120.5	H6A—O6—H6B	100.6
C11—C10—H10A	120.5	H7A—O7—H7B	106.1
N2—C11—C10	124.1 (3)	H7A—O7—H7C	118.2
N2—C11—H11A	117.9	H7B—O7—H7C	109.6
			
C2—N1—C1—C5	−0.7 (4)	C5—C1—C12—N2	179.5 (2)
C2—N1—C1—C12	180.0 (2)	N1—C1—C12—C8	178.7 (2)
C1—N1—C2—C3	0.0 (4)	C5—C1—C12—C8	−0.6 (4)
N1—C2—C3—C4	0.7 (4)	O1—S1—C13—C19	109.1 (2)
C2—C3—C4—C5	−0.7 (4)	O2—S1—C13—C19	−129.0 (2)
C3—C4—C5—C1	0.0 (4)	O3—S1—C13—C19	−10.6 (2)
C3—C4—C5—C6	179.5 (3)	O1—S1—C13—C14	−70.5 (2)
N1—C1—C5—C4	0.7 (4)	O2—S1—C13—C14	51.5 (2)
C12—C1—C5—C4	180.0 (2)	O3—S1—C13—C14	169.9 (2)
N1—C1—C5—C6	−178.8 (2)	C19—C13—C14—C16	2.4 (3)
C12—C1—C5—C6	0.5 (4)	S1—C13—C14—C16	−178.08 (17)
C4—C5—C6—C7	−179.7 (3)	C19—C13—C14—C15	−175.1 (2)
C1—C5—C6—C7	−0.2 (4)	S1—C13—C14—C15	4.4 (3)
C5—C6—C7—C8	0.1 (5)	C16—C14—C15—O4	−171.1 (2)
C6—C7—C8—C9	179.4 (3)	C13—C14—C15—O4	6.4 (4)
C6—C7—C8—C12	−0.3 (4)	C16—C14—C15—C16^i^	5.7 (3)
C12—C8—C9—C10	0.0 (4)	C13—C14—C15—C16^i^	−176.7 (2)
C7—C8—C9—C10	−179.7 (3)	C13—C14—C16—C17	−2.4 (3)
C8—C9—C10—C11	−0.4 (4)	C15—C14—C16—C17	175.3 (2)
C12—N2—C11—C10	0.3 (4)	C13—C14—C16—C15^i^	176.4 (2)
C9—C10—C11—N2	0.3 (5)	C15—C14—C16—C15^i^	−5.9 (4)
C11—N2—C12—C8	−0.8 (4)	C14—C16—C17—C18	0.3 (4)
C11—N2—C12—C1	179.1 (2)	C15^i^—C16—C17—C18	−178.6 (2)
C9—C8—C12—N2	0.7 (4)	C16—C17—C18—C19	1.8 (4)
C7—C8—C12—N2	−179.6 (2)	C17—C18—C19—C13	−1.8 (5)
C9—C8—C12—C1	−179.2 (2)	C14—C13—C19—C18	−0.4 (4)
C7—C8—C12—C1	0.5 (4)	S1—C13—C19—C18	−179.9 (2)
N1—C1—C12—N2	−1.3 (3)		

Symmetry codes: (i) −*x*+2, −*y*, −*z*+1.

### Hydrogen-bond geometry (Å, °)

**Table:**

*D*—H···*A*	*D*—H	H···*A*	*D*···*A*	*D*—H···*A*
N1—H1A···O5	0.87 (3)	1.91 (3)	2.713 (3)	153 (3)
O5—H5A···O2^ii^	0.82	1.97	2.776 (3)	168
O5—H5B···O2	0.82	2.19	2.931 (3)	150
O5—H5B···O4	0.82	2.48	3.131 (3)	138
O6—H6A···O3^iii^	0.82	1.96	2.775 (3)	170
O6—H6B···O1^iv^	0.82	1.92	2.739 (3)	172
O7—H7A···O6	0.82	1.97	2.774 (4)	167
O7—H7B···O7^v^	0.82	2.20	3.021 (6)	178
O7—H7C···O5	0.82	2.48	3.301 (4)	179
C2—H2A···O7	0.93	2.39	3.300 (4)	167
C4—H4A···O1^vi^	0.93	2.58	3.418 (4)	151
C6—H6···O4^vi^	0.93	2.54	3.350 (3)	145
C18—H18···O3^vii^	0.93	2.51	3.357 (4)	152

Symmetry codes: (ii) −*x*+2, −*y*+1, −*z*+1; (iii) *x*, *y*, *z*+1; (iv) −*x*+1, −*y*, −*z*+1; (v) −*x*+2, −*y*+1, −*z*+2; (vi) −*x*+1, −*y*+1, −*z*+1; (vii) −*x*+2, −*y*, −*z*.
